# A lung squamous cell carcinoma-associated membranous nephropathy patient free of tumor and membranous nephropathy after the treatment of surgery and radiochemotherapy following pembrolizumab: A rare case report

**DOI:** 10.1097/MD.0000000000032508

**Published:** 2023-01-20

**Authors:** Feifei Chen, Haiwei Du, Surong Fang

**Affiliations:** a Department of Respiratory Medicine, Nanjing First Hospital, Nanjing Medical University, Nanjing, China; b Burning Rock Biotech, Guangzhou, China.

**Keywords:** case report, lung squamous cell carcinoma, membranous nephropathy, PD-L1, pembrolizumab

## Abstract

Membranous nephropathy (MN) is an autoimmune disease, which is classified into primary and secondary MN. Malignancy-associated MN (M-MN) accounts for about 10% of secondary MN cases. Lung cancer is the most common type of malignancy among M-MN patients. Immune checkpoint inhibitors (ICIs) targeting programmed cell death-1 (PD-1) or programmed cell death ligand-1 (PD-L1) have showed promising efficacy and good safety in many types of solid tumors, including non-small cell lung cancer. To date, whether ICIs could be a treatment option for M-MN patients with PD-L1 expression and or high tumor mutation burden (TMB) level has not been documented.

**Patient concerns:**

A 68-year-old male patient presented with edema of the lower limbs with increased urine foam in August 2018. Biopsy on the right kidney showed MN at stage I with subepithelially localized immune deposits.

**Diagnosis:**

Lung squamous cell carcinoma (LSCC)-associated MN with PD-L1 expression (20%) and high TMB level (26.2 mutations/Mb).

**Interventions:**

The patient received immunosuppressive therapy targeting the initially diagnosed primary MN as first-line treatment plus surgery and radiochemotherapy following pembrolizumab targeting the definitively diagnosed lung cancer as second-line treatment.

**Outcomes:**

The patient benefited from radiochemotherapy following pembrolizumab (lasting more than 38 months) rather than immunosuppressive therapy.

**Lessons:**

Our work suggests that combined ICIs might be an effective treatment option for M-MN patients who harbor PD-L1 expression. Our work highlights that the presence of malignancy should not be neglected at the initial diagnosis of MN.

## 1. Introduction

Membranous nephropathy (MN) is a rare, organ-specific autoimmune disease characterized by the accumulation of immune deposits on the subepithelial (outside) aspect of the glomerular capillary wall, impairing the kidney’s permeability to proteins. It has been documented that MN has an annual incidence of 10 to 12 per million in North America and 2 to 17 per million in Europe with a 2:1 male predominance.^[1]^

It can be subdivided into primary and secondary forms. Primary (or idiopathic) MN accounts for 70% to 80% of MN cases. The remaining cases are secondary MN that can be caused by autoimmune diseases, malignancies, infections, or medications.^[2]^ Despite a common histopathological pattern and clinical presentations, the outcomes and treatment strategies are different between primary and secondary MN. In patients with secondary MN, the underlying cause should be treated. Malignancies are the second most common cause of secondary MN (following autoimmune diseases) that accounts for about 10% of secondary MN cases. Of which, lung cancer was one of most common type of malignances, followed by prostate cancer and hematologic malignancies.^[3]^

Immune checkpoint inhibitors (ICIs) targeting programmed cell death-1 (PD-1) or programmed cell death ligand-1 (PD-L1) have revolutionized the therapeutic landscape of many types of solid tumors with superior efficacy and good safety profile.^[4]^ Whether patients with malignancy-associated MN (M-MN) could benefit from ICIs remains elusive. In this work, we reported that a Chinese MN male associated with lung squamous cell carcinoma (LSCC) who had PD-L1 positive expression and high tumor mutation burden (TMB) level benefited from radiochemotherapy (RCT) following ICI (pembrolizumab). The patient was free of LSCC and MN after treatment of surgery and RCT following pembrolizumab.

## 2. Case story

**Figure 1.**
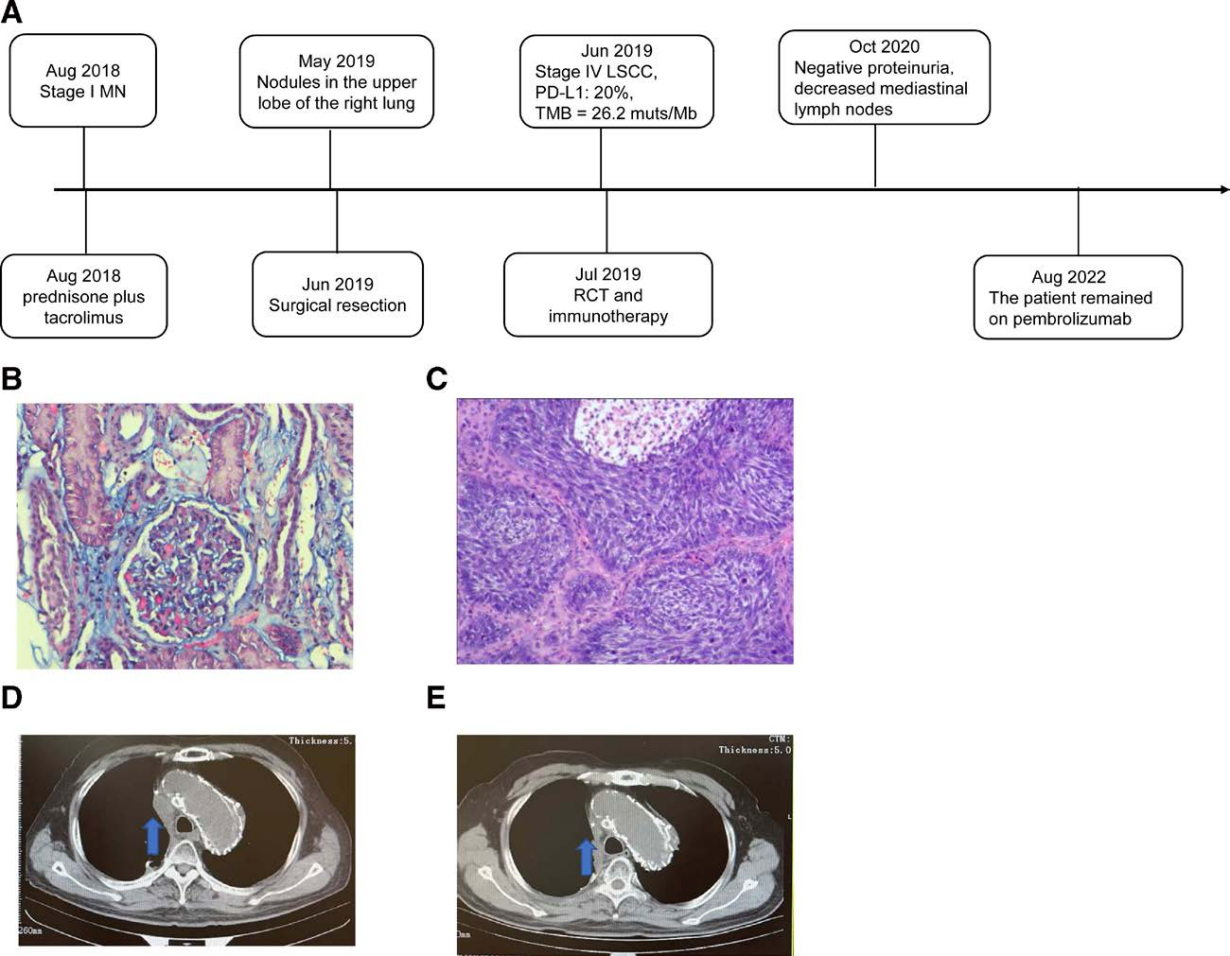
The treatment milestones of the patient. (A) The treatment timeline of the patient; (B) Masson trichrome staining showing subepithelially localized immune deposits (Original magnification, 200×); (C) Hematoxylin-Eosin (HE) staining showing lung cancer (Original magnification, 100×); (D) enlarged mediastinal lymph nodes before chemotherapy; (E) Reduced mediastinal lymph nodes after chemotherapy. LSCC = lung squamous cell carcinoma, MN = membranous nephropathy, muts/Mb = mutations/megabase, PD-L1 = programmed cell death ligand-1, RCT = concurrent local radiotherapy and chemotherapy, TMB = tumor mutation burden.

**Figure 2.**
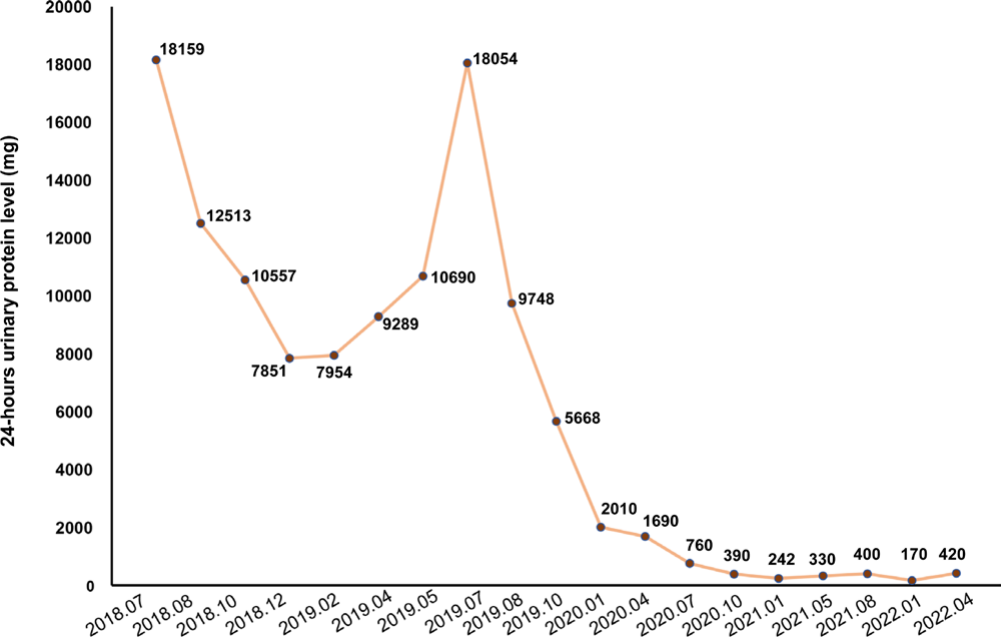
The change of 24-hours urinary protein level of the patient during the treatment period.

A 68-year-old male patient presented with edema of the lower limbs with increased urine foam in August 2018 (Fig. 1A). Biopsy on the right kidney was performed and light microscopy showed MN at stage I with subepithelially localized immune deposits (Fig. 1B). His 24-hours urinary protein level was 18159 mg (Fig. 2). After more than 9 months of taking immunosuppressive therapy tacrolimus plus prednisone, the edema and proteinuria were not improved. The patient then attended our hospital in May 2019 with a 24-hours urinary protein level of 10690 mg (Fig. 2).

Chest computed tomography (CT) showed nodules in the upper lobe of the right lung. The patient then underwent wedge resection of the upper lobe of the right lung and regional lymph node dissection in June 2019. During the operation, due to the observation of the superior vena cava surrounded with lymph nodes mixed with aneurysmal components, the lymph nodes were performed for electrocautery instead of resection. Postoperative pathologic results on the primary lung lesion revealed a medium/poorly differentiated squamous cell carcinoma of the upper lobe of the right lung (2 cm × 1.3 cm × 1 cm) (Fig. 1C) with negative pleural invasion, negative vessel carcinoma embolus, and negative surgical margin. Postoperative pathologic results on resected lymph nodes revealed the presence of metastases in Group 2 (2/3), Group 4 (4/5), and Group 10 (1/1) lymph nodes. Immunohistochemistry staining on the primary lung lesion revealed Napsina (−), TTF-1 (−), P40 (2+), P63 (3+), Syn (−), Cga (−), CD56 (−), and PD-L1 (22C3, 20%+). Meanwhile, captured targeted-based sequencing was performed on the primary lung lesion using a panel consisting of 520 cancer-related genes (Burning Rock Biotech) and revealed a microsatellite stability (MSS) tumor with a high TMB level of 26.2 mutations/Mb and no actionable alterations occurring in *EGFR/ALK/ERBB2/MET/RET/ROS1/BRAF/NTRK/KRAS.* Enlarged mediastinal lymph nodes are shown in Figure 1D. The patient had a performance status of 1. Due to the PD-L1 expression and high TMB level, the patient was subsequently given radiochemotherapy (docetaxel plus carboplatin, RCT) in July 2019 following ICI with a PD-1 inhibitor pembrolizumab at a dose of 200 mg. During the treatment period, negative proteinuria, normal blood albumin, no clinical manifestations of MN, and decreased mediastinal lymph nodes were observed (Fig. 1E). He underwent elevated blood glucose, alopecia, myelosuppression, pulmonary infection, and reflux esophagitis with the treatment of RCT and underwent fatigue with pembrolizumab treatment. At the time of manuscript preparation (Sep 2022), the patient remained on pembrolizumab and was free of LSCC with a progression-free survival of 38 months since surgical resection and was free of MN lasting more than 23 months.

## 3. Discussion

To the best of our knowledge, this is the first study to report the treatment efficacies of ICIs in M-MN patients with PD-L1 expression. In this study, the onset age of the MN patient with LSCC was 68 years, which was consistent with the data from a previous meta-analysis study indicating that the mean age of MN patients with cancer was 67 ± 7 years.^[3]^ To date, the molecular mechanisms of MN with concurrent cancer remain elusive. Beck^[5]^ proposed four possible mechanisms: (1) it may be that an antibody is produced in response to tumor antigen similar to endogenous podocellular antigen immunity, leading to the formation of in situ immune complexes; (2)tumor antigens shed from the circulating immune complex are subsequently deposited on the glomerular capillary wall; (3) circulating antibodies may also act on colonized subepithelial tumor antigens; or (4) an external process such as viral infection or an underlying abnormal immune response may be cancer and MN.

At present, there is no recognized diagnostic criterion for M-MN. The following diagnostic basis are commonly used: the tumor and MN occurs within 1 year; the tumor and MN are present at the same time or the tumor is found before MN; no other secondary causes of MN are found.^[6]^ In this study, although MN was found before LSCC, the patient should be diagnosed with M-MN rather than primary MN due to the following reasons. First, lung cancer is an insidious disease until the disease spreads widely, resulting in no obvious symptoms related to lung cancer before the MN-related symptoms in the patient. Second, the patient benefited from RCT following ICI targeting lung cancer rather than immunosuppressive therapy targeting primary MN. These findings suggest that the presence of malignancy should not be neglected at the initial diagnosis of MN. A definitive diagnosis of MN with primary MN or secondary MN (especially M-MN) enables patients to benefit from appropriate treatment strategies.

Pembrolizumab, a human monoclonal anti-PD-1 antibody, inhibits the interaction of PD-1 with PD-L1/ programmed cell death ligand-2 (PD-L2), thereby promoting an immunologic response by T cells against tumor cells. National Comprehensive Cancer Network (NCCN) guidelines recommend carboplatin + (paclitaxel or albumin-bound paclitaxel) + pembrolizumab as first-line treatment for advanced or metastatic LSCC patients with PD-L1 tumor proportion score ≥1% and a performance status of 0 to 2.^[7]^ Besides PD-L1, TMB is a potential immunotherapy biomarker. Some previous studies have revealed the enhanced treatment response rate and survival outcome in patients with a high level of tissue-based TMB who receive ICIs in lung cancer.^[8]^ In this study, the MN patient with stage IV LSCC had high PD-L1 expression (20%) and high TMB level (26.2 mutations/Mb), which indicated that the patient might benefit from ICIs. As expected, he had negative proteinuria and decreased mediastinal lymph nodes at nine months after the start of treatment with concurrent local radiotherapy and chemotherapy (docetaxel plus carboplatin) following pembrolizumab. At the time of manuscript preparation, the patient remained on pembrolizumab as maintenance therapy and had a PFS of 38 months. Chen et al have reported a metastatic non-small cell lung cancer patient who developed MN after ICIs therapy.^[9]^ Kim have described a case of nephrotic syndrome relapse in a patient with a history of MN during PD-L1 inhibitor durvalumab therapy for non-small cell lung cancer.^[10]^ These findings indicate that there is a risk of M-MN patients with MN relapse or nephrotic syndrome after ICIs therapy for cancers, which should be noted in the clinical practice of M-MN.

There are some limitations associated with this study. First, only one patient was included. Clinical trials or large cohort studies are warranted to investigate the efficacy and safety of ICIs in patients with M-MN. Second, underlying mechanisms of LSCC-associated MN were not investigated in this work. Lifting the veil referring to the underlying mechanisms of cancer-associated MN is warranted to provide novel diagnostic and therapeutical perspectives of cancer-associated MN in further work.

Our work suggests that RCT with sequential ICIs might be an efficacious treatment option for MN patients with advanced/metastatic lung cancer who had PD-L1 expression and or a high TMB level. Our work also highlights that malignancy should not be neglected at the initial diagnosis of MN. For clinically diagnosed MN patients, a general check is required to find out the cause of MN due to the fact that primary MN and secondary MN patients are treated with different regimens in clinical practice.

## Author contributions

**Conceptualization:** Surong Fang.

**Data curation:** Feifei Chen.

**Formal analysis:** Feifei Chen, Surong Fang.

**Writing – original draft:** Feifei Chen, Haiwei Du, Surong Fang.

**Writing – review & editing:** Feifei Chen, Haiwei Du, Surong Fang.
